# Analysis of Genetic Variation in the Bovine *SLC11A1* Gene, Its Influence on the Expression of NRAMP1 and Potential Association With Resistance to Bovine Tuberculosis

**DOI:** 10.3389/fmicb.2020.01420

**Published:** 2020-06-30

**Authors:** Angela Holder, Rachel Garty, Charlotte Elder, Paula Mesnard, Celine Laquerbe, Marie-Christine Bartens, Mazdak Salavati, Muhammad Zubair Shabbir, Thomas Tzelos, Timothy Connelly, Bernardo Villarreal-Ramos, Dirk Werling

**Affiliations:** ^1^Department of Pathobiology and Population Sciences, Royal Veterinary College, Hertfordshire, United Kingdom; ^2^EPLEFPA Agricampus La Roque, Rodez, France; ^3^The Roslin Institute, The University of Edinburgh, Midlothian, United Kingdom; ^4^University of Veterinary and Animal Sciences, Lahore, Pakistan; ^5^Institute of Biological Environmental and Rural Sciences (IBERS), Aberystwyth University, Aberystwyth, United Kingdom; ^6^APHA, Webybridge, United Kingdom

**Keywords:** SLC11A1, NRAMP1, bovine tuberculosis, genetics, cattle breeds

## Abstract

Bovine tuberculosis (bTB), caused by *Mycobacterium bovis*, is a chronic zoonotic disease where host genetics is thought to contribute to susceptibility or resistance. One of the genes implicated is the *SLC11A1* gene, that encodes for the natural resistance-associated macrophage protein 1 (NRAMP1). The aim of this study was to identify *SLC11A1* polymorphisms and to investigate any resulting functional differences in NRAMP1 expression that might be correlated with resistance/susceptibility to *M. bovis* infection. Sequencing of the *SLC11A1* gene in cDNA isolated from Brown Swiss, Holstein Friesian, and Sahiwal cattle identified five single nucleotide polymorphisms (SNPs) in the coding region, but only one of these (SNP4, c.1066C>G, rs109453173) was present in all three cattle breeds and therefore warranted further investigation. Additionally, variations of 10, 11, and 12 GT repeats were identified in a microsatellite (MS1) in the *SLC11A1* 3′UTR. Measurement of NRAMP1 expression in bovine macrophages by ELISA showed no differences between cells generated from the different breeds. Furthermore, variations in the length of the MS1 microsatellite did not impact on NRAMP1 protein expression as analyzed by luciferase reporter assay. However, further analysis of the ELISA data identified that the presence of the alternative G allele at SNP4 was associated with increased expression of NRAMP1 in bovine macrophages. Since NRAMP1 has been shown to influence the survival of intracellular pathogens such as *M. bovis* through the sequestering of iron, it is possible that cattle expressing the alternative G allele might have an increased resistance to bTB through increased NRAMP1 expression in their macrophages.

## Introduction

Bovine tuberculosis (bTB), caused by *Mycobacterium bovis*, is a chronic disease of worldwide importance due to the major economic and zoonotic implications associated with it. In the United Kingdom, approximately 30,000 cattle are slaughtered each year due to bTB at a cost of around £100 million (UK Government Bovine TB, 2020), while the annual cost of bTB to worldwide agricultural is estimated to be around three billion US dollars (Allen et al., 2010). However, bTB does not only affect cattle, it also represents a risk to human health, with 10–15% of human TB cases worldwide being attributed to an *M. bovis* infection (Michel et al., 2010).

Recent research into controlling the spread of bTB has focused on improving diagnostic testing (Schiller et al., 2010) as well as the development of suitable vaccines for livestock and/or wildlife reservoirs with the ability to differentiate between vaccinated and infected animals (Chambers et al., 2014; Buddle et al., 2018). However, it has been observed that different cattle breeds vary in their ability to clear bacterial pathogens, suggesting that the host’s genetics play an important role in generating a potentially protective immune response (Rupp and Boichard, 2003; Allen et al., 2010; le Roex et al., 2013). Indeed, a comparison between breeds showed that Simmental and Brown Swiss (BS) cattle demonstrated a lower clinical mastitis frequency compared to Holstein-Friesian (HF) cattle (Rupp and Boichard, 2003), while studies in Ethiopia identified a lower incidence rate as well as reduced severity of *M. bovis* infections in zebu cattle (*Bos indicus*) compared to HF cattle (*Bos taurus*) (Ameni et al., 2007; Vordermeier et al., 2012). Additionally, monocyte-derived macrophages (MDM) from BS cattle produced more reactive oxygen species and showed increased phagocytic and microbicidal capabilities compared to MDM generated from HF cattle, suggesting that BS cattle might be more effective in dealing with bacterial pathogens compared HF cattle (Gibson et al., 2016). Therefore, the identification of genetic traits impacting on susceptibility or resistance to bTB might offer alternative ways of controlling the disease through selective breeding (Warner et al., 1987; Allen et al., 2010; le Roex et al., 2013). Indeed, genomic regions and candidate genes were recently identified in HF cattle which may provide an opportunity to further understand pathways critical to cattle susceptibility to bTB (Raphaka et al., 2017).

The genome-wide association study (GWAS) approach has provided a tool to identify chromosomal regions in the bovine genome that are associated with susceptibility to bTB in order to try and identify specific candidate genes for further analysis (Finlay et al., 2012; Bermingham et al., 2014; Raphaka et al., 2017; Ring et al., 2019). However, one of the potentially most promising candidate gene was identified in murine studies performed over 30 years ago. Here, a locus (originally designated Ity/Lsh/Bcg) was identified that was responsible for innate resistance to the intracellular pathogens *Salmonella typhimurium*, *Leishmania donovani* and *M. bovis* BCG (Plant et al., 1982; Skamene et al., 1982). This locus has since been identified as *SLC11A1*, a member of the solute carrier family 11, that encodes for the natural resistance-associated macrophage protein 1 (NRAMP1). NRAMP1 is a multi-pass membrane protein that regulates macrophage activation in infectious and autoimmune diseases by acting as a transporter protein for protons, iron and other divalent cations (Blackwell et al., 2000).

A previous study has identified single nucleotide polymorphisms (SNPs) in the bovine *SLC11A1* gene that are associated with susceptibility to bTB (Cheng et al., 2015). A study in Taiwanese HF cattle identified SNPs in exon 4 and intron 4 that were associated with enhanced susceptibility to bTB (Cheng et al., 2015), while in a Chinese HF cattle population, associations with bTB susceptibility/resistance were found with SNPs in exon 11 and introns 5 and 9 (Liu et al., 2017). Additionally polymorphisms in the 3′UTR of the bovine *SLC11A1* gene, that have been shown to cause changes to the lengths of two microsatellites (MS1 and MS2), have been identified in various breeds of cattle (Paixao et al., 2006; Vazquez-Flores et al., 2006; Hasenauer et al., 2013). Interestingly, an increase in the length of the MS1 microsatellite has been described to reduce traits indicative of bTB infection in African zebu cattle (Kadarmideen et al., 2011). In contrast, no association between the MS1 microsatellite and response to the tuberculin skin test was found in a study performed using HF and Jersey cattle in Argentina (Hasenauer et al., 2018). Furthermore, no associations between the MS2 microsatellite and susceptibility/resistance to bTB have currently been identified (Barthel et al., 2000; Hasenauer et al., 2018). Therefore, in the present study we characterized polymorphisms in the bovine *SLC11A1* gene in two *B. taurus* breeds, BS and HF, both present in the United Kingdom, and compared these to those found in Sahiwal cattle, a *B. indicus* breed. Furthermore, functional differences in NRAMP1 protein expression resulting from the genetic variation identified were subsequently investigated in order to potentially explain some of the previously identified breed differences seen regarding susceptibility to bTB.

## Materials and Methods

### Samples

Peripheral blood mononuclear cells (PBMCs) were purified by density gradient centrifugation (Lymphoprep, STEMCELL technologies) using whole blood from 15 BS and 15 HF cattle reared at the Royal Veterinary College (Hatfield, United Kingdom) and Cancourt Farm (Swindon, United Kingdom), and sampled under Home Office license (PPL7009059). RNA was extracted from the PBMCs using the RNeasy Mini Kit (Qiagen) and cDNA was synthesized using the iScript cDNA Synthesis Kit (Bio-Rad). Complementary DNA samples from 15 Sahiwal cattle, reared on farms in Pakistan, were a provided by The Roslin Institute (University of Edinburgh, Edinburgh, United Kingdom). The sample size used in this study was limited by aforementioned Home Office license.

### Sequencing and Analysis of the Bovine *SLC11A1* Gene

All 45 samples were used for sequencing. Specific primers to amplify the coding region of the bovine *SLC11A1* gene were designed using publicly available sequence information^1^ and Primer Blast^2^ (Supplementary Table S1). Primers to amplify a specific region of the *SLC11A1* 3′UTR containing a previously described microsatellite [MS1, c.1647+61GT(10_13)] were taken from the current literature (Hasenauer et al., 2013; Supplementary Table 1).

PCR reactions were carried out using Phusion High-Fidelity Master Mix (Thermo Fisher Scientific). PCRs were performed in 50 μl reactions containing 25 μl 2× Phusion Master Mix, 3 μl cDNA and 2.5 μl of each primer (forward and reverse, 10 μM). Due to the GC rich nature of the *SLC11A1* gene 3% DMSO was included in PCR reactions used to amplify the coding region. Thermocycling conditions for the coding region consisted of an initial denaturation step at 98°C for 30 s, followed by 36 cycles of 98°C for 10 s (denaturation), 65.5°C for 30 s (annealing) and 72°C for 30 s (extension), with a final extension step at 72°C for 10 min (Mastercycler Pro S, Eppendorf). For amplification of the MS1 microsatellite the annealing and elongation steps were changed to 62°C for 20 s and 72°C for 15 s, respectively. The PCR products generated were separated by agarose gel electrophoresis, purified using the QIAquick Gel Extraction Kit (Qiagen), and then submitted for sequencing (MRC PPU DNA Sequencing Service, Dundee University).

Single nucleotide polymorphisms were identified using CLC Main Workbench version 6.0.2 (CLCbio, Aarhus, Denmark). Allele and genotype frequencies were calculated for each SNP and compared between cattle breeds using a Fisher Exact Test^3^. Phylogenetic analysis was performed in CLC Main Workbench using the coding region genotypes identified in the study and the bovine reference sequence^4^. Heterozygous loci were labeled using IUPAC ambiguity codes. A maximum likelihood phylogenetic tree was constructed using a Jukes Cantor model and 100 bootstrap iterations.

### Measurement of NRAMP1 Protein in Bovine Monocyte-Derived Macrophages by ELISA

Monocytes were isolated from PBMCs (11 BS and 12 HF) by magnetic activated cell sorting (MACS). Briefly, PBMCs were incubated with paramagnetic beads labeled with an anti-CD14 antibody (MicroBeads, Miltenyi Biotec) and CD14^+^ monocytes were isolated by positive selection on LS columns in a MidiMACS magnetic separator (Miltenyi Biotech) as described before (Gibson et al., 2016). Monocytes were cultured at approximately 1 × 10^6^ cells/ml in RPMI 1640 medium containing GlutaMAX-1 (Gibco) supplemented with 10% Fetal Bovine Serum (FBS, Sigma-Aldrich) and 1% Penicillin-Streptomycin (PenStrep, Gibco). Cells were differentiated into MDM by culturing them for 6 days in the presence of 20 ng/ml recombinant bovine (rbo)M-CSF (Kingfisher Biotech). After 6 days in culture, MDM were harvested using Accutase (Gibco), washed twice in PBS and counted using an automated cell counter (TC20, Bio-Rad). MDM were lysed subsequently by freeze-thawing three times, before being centrifuged at 1000 × *g* for 15 min to remove any debris. Cell lysates were assayed for NRAMP1 concentrations using a bovine specific NRAMP1 ELISA kit (BlueGene) according to the manufacturer’s instructions. Standards and samples were assayed in duplicate. A standard curve was generated using optical density (OD) 450 nm values obtained for the standards provided, and the concentration of NRAMP1 protein in the cell lysates was interpolated from this. Subsequently, NRAMP1 concentrations were normalized for cell number.

### Dual-Glo miRNA Target Luciferase Reporter Assay

A 117 bp region of the *SLC11A1* 3′UTR containing the MS1 microsatellite was amplified by PCR (see Supplementary Table 1 for primers) using cDNA samples from cows representing the three different lengths of MS1 identified in the study (10, 11, and 12 repeats of GTs). PCR products were purified by gel extraction (QIAquick Gel Extraction Kit, Qiagen) and ligated into a dual-luciferase miRNA target expression vector (pmirGLO, Promega) using the *PmeI* and *XbaI* restriction sites located in the multiple cloning site of the vector and T4 DNA ligase (Thermo Fisher Scientific).

Two different cell lines, chinese hamster ovary (CHO) and murine macrophage-like RAW264.7 cells were transfected with recombinant pmirGLO constructs. CHO cells were plated at 1 × 10^5^ cells/well in 24 well plates in Minimal Essential Medium (MEM) supplemented with 10% FBS, 1% non-essential amino acid solution (Sigma-Aldrich), 1% GlutaMAX-1, 1% PenStrep (Gibco), and cultured overnight until they reached 70–90% confluency. At this point, CHO cells were transfected with 800 ng plasmid DNA using Turbofect (Thermo Fisher Scientific) according to the manufacturer’s instructions. RAW264.7 cells were plated at the same density but cultured in RPMI-1640 medium containing GlutaMAX-1 (Gibco), supplemented 10% FBS (Sigma-Aldrich), 1 mM Sodium Pyruvate and 1% PenStrep (Gibco). They were transfected with 500 ng plasmid DNA using Lipofectamine 3000 (Invitrogen) according to the manufacturer’s instructions. Untransfected cells were used as a negative control.

Twenty-four hours after transfection, cells were assayed for both firefly and renilla luciferase activity, using the Dual-Glo Luciferase Assay System (Promega). The cells were lysed in 200 μl 1% Triton ×100 (Sigma-Aldrich) and 50 μl was transferred to triplicate wells on a 96 well white walled plate (Corning). Fifty microliters Dual-Glo Luciferase Reagent was added to each well as a substrate for firefly luciferase. After 15 min incubation on a rotating platform luciferase activity was measured using a luminometer (SpectraMax L, Molecular Devices Ltd.). Next, 50 μl Dual-Glo Stop & Glo Reagent was added, and after another 15 min incubation luminescence was measured for a second time to obtain a reading for renilla luciferase activity. Firefly luciferase activity was then normalized against renilla luciferase activity.

### Statistical Analysis

Groups were compared using either an unpaired *t*-test or one-way ANOVA following a Shapiro–Wilk test to confirm normality. Significant differences were donated as followed in the figures: ^∗^*p* < 0.05; ^∗∗^*p* < 0.01; ^∗∗∗^*p* < 0.001.

## Results

### Analysis of Polymorphisms in the Bovine *SLC11A1* Gene

Sequencing of the *SLC11A1* coding region in cDNA generated from BS, HF, and Sahiwal cattle identified five SNPs. One of these was synonymous while the remaining four resulted in amino acid substitutions (Supplementary Table 2).

The *SLC11A1* coding region in BS and HF cattle appeared to contain less genetic variation compared to Sahiwal cattle (Table 1 and Supplementary Table 3). Four out of the five SNPs identified occurred at significantly higher frequencies in Sahiwal cattle compared to BS and HF cattle. In fact, SNP1 and SNP5 (c.87A>G, rs109614179 and c.1592G>C, rs110347562) were found to be present only in the Sahiwal cattle. SNP2 (c.650C>T, rs109915208) was only found in HF cattle, although it was present at quite a low frequency (10%). SNP4 (c.1066C>G, rs109453173) was the only one identified as having an alternative allele frequency of >10% in all three cattle breeds, suggesting it has the potential to be a significantly polymorphic locus. None of the SNPs were found to occur at significantly different frequencies between the BS and HF cattle.

**TABLE 1:** Analysis of *SLC11A1* coding region SNPs in Brown Swiss, Holstein-Friesian and Sahiwal cattle.

SNP ID	Cattle breed	Allele frequency (%)		*P*-value	Genotype frequency (%)			*P*-value
				
		Reference	Alternative		Reference	Heterozygous	Alternative	
SNP1	BS	30 (100)	0		15 (100)	0	0	
c.87A>G	HF	30 (100)	0	1	15 (100)	0	0	1
rs109614179	SW	22 (73.3)	8 (26.7)	**0.0002**	9 (60)	4 (26.7)	2 (13.3)	**0.0018**
SNP2	BS	30 (100)	0		15 (100)	0	0	
c.650C>T	HF	27 (90)	3 (10)	0.237	13 (86.6)	1 (6.7)	1 (6.7)	0.483
rs109915208	SW	30 (100)	0	0.104	15 (100)	0	0	0.318
SNP3	BS	29 (96.7)	1 (3.3)		14 (93.3)	1 (6.7)	0	
c.961G>A	HF	30 (100)	0	1	15 (100)	0	0	1
rs109551090	SW	21 (70)	9 (30)	**0.0002**	8 (53.4)	5 (33.3)	2 (13.3)	**0.0038**
SNP4	BS	23 (76.7)	7 (23.3)		9 (60)	5 (33.3)	1 (6.7)	
c.1066C>G	HF	26 (86.7)	4 (13.3)	0.506	12 (80)	2 (13.3)	1 (6.7)	0.682
rs109453173	SW	17 (56.7)	13 (43.3)	**0.0421**	6 (40)	5 (33.3)	4 (26.7)	0.188
SNP5	BS	30 (100)	0		15 (100)	0	0	
c.1592G>C	HF	30 (100)	0	1	15 (100)	0	0	1
rs110347562	SW	25 (83.3)	5 (16.7)	**0.0097**	11 (73.3)	3 (10)	1 (6.7)	**0.018**

*Genotype and allele frequencies were compared between cattle breeds for each SNP. *P*-values were generated using Fisher’s exact test. Comparisons were made between Brown Swiss and Holstein-Friesian cattle (upper *P*-values) and between all three cattle breeds (lower *P*-values). Significant *P*-values are shown in bold. BS, Brown Swiss; HF, Holstein-Friesian; SW, Sahiwal.*

Analysis of the MS1 *SLC11A1* 3’UTR microsatellite [c.1647+61GT(10_13)] in BS, HF, and Sahiwal cattle found very little difference in the frequency of the different lengths of this GTn polymorphism between the three cattle breeds (Table 2 and Supplementary Table 3).

**TABLE 2:** Analysis of the MS1 *SLC11A1* 3′UTR microsatellite in Brown Swiss, Holstein-Friesian, and Sahiwal cattle.

Cattle breed	Allele frequency (%)			*P*-value	Genotype frequency (%)						*P*-value
			
	10	11	12		10/10	10/11	10/12	11/11	11/12	12/12	
BS	3 (10)	12 (40)	15 (50)		1 (6.7)	0	1 (6.7)	0	12 (80)	1 (6.7)	
HF	2 (6.7)	12 (40)	16 (53.3)	1	1 (6.7)	0	0	0	12 (80)	2 (13.3)	0.999
SW	2 (6.6)	14 (46.7)	14 (46.7)	0.966	0	1 (6.7)	1 (6.7)	0	13 (86.6)	0	0.889

*Genotype and allele frequencies were compared between cattle breeds for the different numbers of GT repeats in the microsatellite. *P*-values were generated using Fisher’s exact test. Comparisons were made between Brown Swiss and Holstein-Friesian cattle (upper *P*-values) and between all three cattle breeds (lower *P*-values). Significant *P*-values are shown in bold. BS, Brown Swiss; HF, Holstein-Friesian; SW, Sahiwal.*

Phylogenetic analysis was used to assess evolutionary similarities within the coding region of the *SLC11A1* gene between BS, HF and Sahiwal cattle (Supplementary Figure 1). The bovine reference sequence, which originates from a Hereford bull, was included in the analysis for comparative reason. The analysis shows that the sequences cluster into three main groups, with clusters in two of these groups being independent of cattle breed, and only Sahiwal cattle genotypes being present in the third group. This is unusual as sequences from different sub-species, such as the *B. taurus* and *B. indicus* cattle, would be expected to diverge into separate clades on a molecular phylogenetic tree.

### NRAMP1 Protein Expression in Bovine Macrophages Is Associated With a Specific Polymorphism in the Coding Region of the *SLC11A1* Gene

**FIGURE 1:**
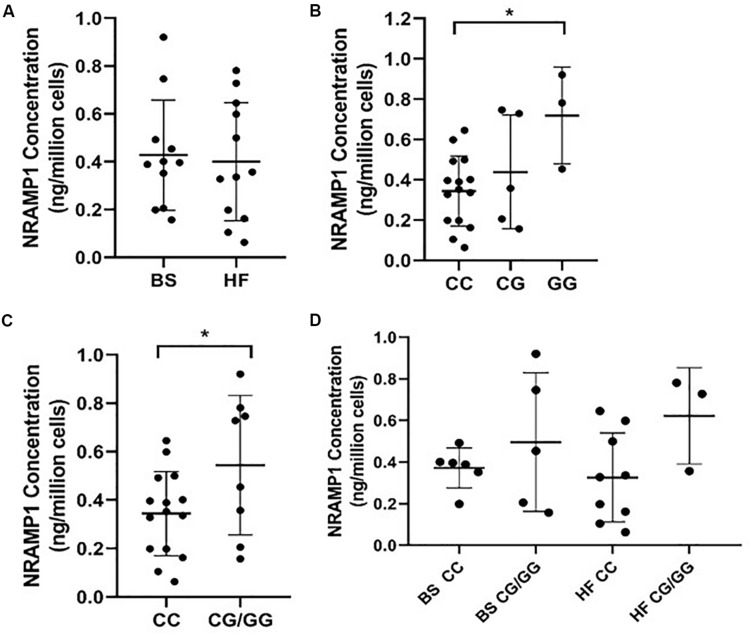
NRAMP1 expression in bovine MDMs is associated with *SLC11A1* SNP4 (c.1066C>G). NRAMP1 protein was measured in cell lysates by ELISA and concentrations were normalized for cell number. Each sample is represented by a filled circle on the dot plots. Samples are grouped according to **(A)** cattle breed **(B)** *SLC11A1* SNP4 genotype, **(C)** the presence or absence of the minor G allele, **(D)** cattle breed and the presence or absence of the minor G allele. The mean and SD of each group are represented by horizontal lines. *P*-values were calculated using **(B)** a one-way ANOVA with *post hoc* Tukey HSD test and **(C)** an unpaired *t*-test. *P-value < 0.05. BS, Brown Swiss; HF, Holstein-Friesian.

As NRAMP1 protein concentration in MDMs generated from BS and HF cattle did not show any significant difference as analyzed by ELISA (Figure 1A), we assessed the impact of *SLC11A1* SNP4 (c.1066C>G, rs109453173) instead. Analysis of NRAMP1 expression in MDMs from cows possessing each of the three different *SLC11A1* SNP 4 genotypes (CC, CG, GG) resulted in significantly higher NRAMP1 expression in cells from the alternative GG genotype cows compared to the reference CC genotype (*p* = 0.025) (Figure 1B). Similarly, MDMs from cows with the heterozygous cows CG genotype also had increased NRAMP1 expression compared to the reference CC genotype, although this difference did not reach significance. This suggests the G allele has an additive effect on NRAMP1 expression. Combining the results for the CG and GG genotype revealed a significantly increased NRAMP1 expression in MDMs generated from cows carrying the alternative G allele (*p* = 0.049; Figure 1C). The influence of the alternative G allele at SNP4 was then analyzed separately by breed (Figure 1D). Although NRAMP1 expression is clearly higher in MDMs with the alternative G allele in both, BS and HF, the differences were not significant. This is likely to be the result of reduced numbers in each of the groups compared to when the breeds were pooled together for the previous analysis.

### The *SLC11A1* 3′UTR MS1 Microsatellite Does Not Appear to Influence Post-transcriptional Regulation in Macrophages

A dual luciferase reporter assay was employed to assess the possible functional effect of the MS1 microsatellite in the *SLC11A1* 3′UTR. Recombinant plasmid DNA constructs were designed that contained a 117 bp region of the *SLC11A1* 3′UTR, representing the three lengths of MS1 microsatellite identified in this study (10, 11, or 12 GTs), located downstream of the firefly luciferase coding sequence and within its 3′UTR.

**FIGURE 2:**
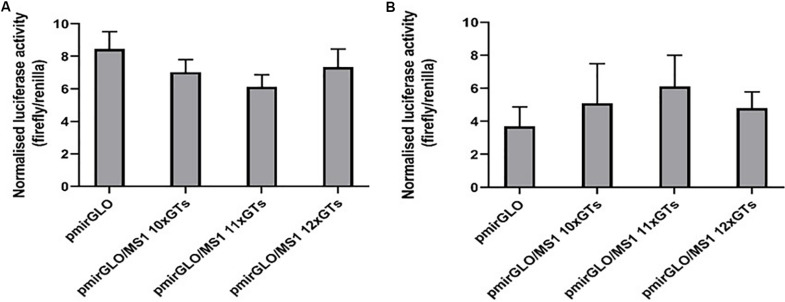
Different lengths of the *SLC11A1* 3′UTR MS1 microsatellite do not appear to influence post-transcriptional regulation. **(A)** CHO and **(B)** RAW 264.7 cells were transfected with pmirGLO constructs containing different lengths of the MS1 microsatellite. After 24 h renilla and firefly luciferase activity was measured using the Dual-GLO luciferase assay system. Normalized luciferase activity (firefly/renilla) was then calculated for each construct. Results are the mean ± SEM of three experiments.

Chinese hamster ovary and RAW264.7 cells transfected with recombinant pmirGLO constructs were assayed for both renilla luciferase activity, to estimate transfection efficiency, and firefly luciferase activity, which is the primary reporter gene (Supplementary Figure 2). Firefly luciferase activity was then normalized against renilla luciferase activity and the constructs containing the different lengths of microsatellite compared to the empty pmirGLO plasmid (Figure 2 and Supplementary Figure 2).

In CHO cells, expression of the reporter protein appeared to be reduced in cells transfected with the pmirGLO/MS1 11×GTs construct, compared to both the empty pmirGLO plasmid and the constructs containing lengths of 10× and 12×GTs (Figure 2A). However, this difference was not found to be significant. In contrast, in murine RAW264.7 cells transfection with the pmirGLO/MS1 11×GTs construct gave higher expression of the reporter protein compared to the others, but again this difference was not significant (Figure 2B). This suggests that differences in the number of GT repeats in the *SLC11A1* MS1 microsatellite do not appear to significantly impact post-transcriptional regulation and that this may vary depending on the cell line used.

## Discussion

This study was designed to identify potential polymorphisms in the bovine *SLC11A1* gene and examine the influence these might have on the expression of NRAMP1, a protein that plays an important role in dealing with intracellular pathogens, and in particular *M. bovis* (Allen et al., 2010). Sequencing of the *SLC11A1* coding region in cDNA from BS, HF and Sahiwal cattle identified five SNPs. All these SNPs have been identified previously and their details cataloged in the relevant databases^5^. For the present study, the non-synonymous polymorphism located in exon 11 (SNP4, c.1066C>G, rs109453173) was of special interest, as this was the only SNP present in all three cattle breeds. The presence of the alternative G allele for this SNP was associated with higher expression of NRAMP1 in MDM. Sequencing of the *SLC11A1* 3′UTR confirmed the presence of a microsatellite [MS1, c.1647+61GT(10_13)], with variable numbers of GT repeats in all three cattle breeds, but none of these seemed to significantly impact on protein expression.

Four of the five SNPs identified in the bovine SLC11A1 coding region were found to be non-synonymous and therefore have the potential to alter protein function. Sequence alignments, hydropathy profiling and epitope mapping studies have predicted the structure of mammalian NRAMP1 to consist of 12 hydrophobic trans-membrane domains (TMD) joined by alternating extracellular and cytoplasmic loops (Nevo and Nelson, 2006; Cellier et al., 2007; Montalbetti et al., 2013), and analysis performed using the structure of bovine NRAMP1 suggests that this is the same in cattle (Feng et al., 1996; Triantaphyllopoulos et al., 2019). SNP2 and SNP3 (c.650C>T, rs109915208 and c.961G>A, rs109551090), identified in this study resulted in amino acid changes (alanine to valine, aspartic acid to asparagine) in the third and fourth extracellular loops, respectively. SNP4 (c.1066C>G, rs109453173) resulted in proline to alanine substitution in TDM8, and SNP5 (c.1592G>C, rs110347562) resulted in an arginine to proline change in the c-terminus of the protein. As there is limited information regarding the function of NRAMP1 available, the effects these changes might have are difficult to assess. Mutation studies on another NRAMP family member (DMT1/NRAMP2) have shown that the first extracellular loop is involved in metal ion binding (Cohen et al., 2003), while TMD 2, 4, and 6 are essential for metal transport (Lam-Yuk-Tseung et al., 2003; Nevo and Nelson, 2004; Nevo, 2008). Since none of the polymorphisms identified in this study are located in these critical regions, it is difficult to assume how severe their effects might be. However, molecular modeling of the structure of bovine NRAMP1 (Triantaphyllopoulos et al., 2019) using the crystal structures of three different prokaryotic NRAMP orthologs (ScaDMT, DraNramp, and EcoDMT) (Ehrnstorfer et al., 2014; Bozzi et al., 2016; Ehrnstorfer et al., 2017) as comparison suggested that TMD 1, 2, 6, and 7 form a bundle of α-helices involved in ion transportation, while TMD 3, 4, 5, 8, 9, and 10 form a scaffold that supports this bundle. Therefore, it is possible that the change in TDM 8 caused by SNP4 potentially alters the structure of the scaffold and therefore the function of bovine NRAMP1.

A comparison of the *SLC11A1* coding region between the three cattle breeds used in this study revealed that the Sahiwal cattle (*B. indicus*) showed a higher genetic variation compared to BS and HF cattle (*B. taurus*), with four out of the five SNP alleles occurring at significantly higher frequencies in the Sahiwal cattle. Despite this observed genetic variation, the molecular phylogenetic analysis unexpectedly demonstrated no divergence between the two cattle sub-species. This might indicate that certain polymorphisms in the bovine *SLC11A1* gene are being maintained in cattle populations through balancing selection, a suggestion that has also been described for the human *SLC11A1* gene (Blackwell and Searle, 1999; Searle and Blackwell, 1999; Sanjeevi et al., 2000); however, a larger study across global cattle populations would be required to confirm this. Therefore, differences in the frequency of alleles at maintained polymorphic sites within the *SLC11A1* gene could be contributing to previously described differences in resistance to *M. bovis* between *B. indicus* and *B. taurus* breeds (Ameni et al., 2007; Vordermeier et al., 2012). Previous studies also suggested that BS cattle are more resistant to bacterial pathogens compared to HF cattle (Rupp and Boichard, 2003; Gibson et al., 2016). However, our current work does not allow for an assumption to be made whether this is in part due to SNPs in NRAMP1, as no significant differences in *SLC11A1* SNP allele frequencies were observed between these two cattle breeds, potentially due to the small sample size. SNP4 (c.1066C>G, rs109453173) was the only polymorphism present in all three cattle breeds and therefore warranted further investigation. The frequency of the alternative allele at this SNP was found to be highest in the Sahiwal cattle, thought to be more resistant to bTB (Ameni et al., 2007; Vordermeier et al., 2012), and lowest in HF cattle, which seem to be more susceptible to bTB (Ameni et al., 2007; Vordermeier et al., 2012). Interestingly, this SNP has previously been associated with resistance/susceptibility to bTB in Chinese Holstein cattle, where the presence of the alternative allele in the CG and GG genotypes appeared to confer resistance to *M. bovis* infection (Liu et al., 2017).

Cellular lysates from MDMs did not show any differences in the NRAMP1 concentration between MDM generated from BS or HF cattle. This result is consistent with our analysis regarding the *SLC11A1* gene polymorphisms, that also showed no significant difference between these two cattle breeds. Unfortunately, due to import restriction based on the occurrence of Foot-and-Mouth Disease in Pakistan, PBMCs from Sahiwal cattle were not available for this study. Since SNP4 was the only polymorphism present in all three cattle breeds, we compared the impact between three different genotypes (CC, CG, and GG) identified for this polymorphism on MDM NRAMP1 concentration. Combining NRAMP1 values obtained for carriers of the CG and GG genotypes showed that the presence of the G allele was significantly associated with increased NRAMP1 expression, compared to the CC genotype. Previous studies showed that NRAMP1 has the ability to lower the phagosomal iron concentrations, thus impacting on survival and growth of intracellular pathogens, such as *M. bovis*, by depriving them of this essential nutrient (Forbes and Gros, 2001; Cellier et al., 2007). Therefore, it is tempting to speculate that cattle carrying the alternative G allele have an increased resistance to bTB through increased NRAMP1 expression in their macrophages, which would be supported by the results published recently in Chinese HF cattle (Liu et al., 2017). In this study, the authors indeed describe an increased resistance to *M. bovis* in cattle carrying the alternative G allele. In addition, we found the frequency of this G allele to be highest in DNA of *B. indicus* Sahiwal cattle, which also seem to have an increased bTB resistance (Ameni et al., 2007; Vordermeier et al., 2012).

A comparison of the number of GT repeats in the MS1 microsatellite present in the 3′UTR of *SLC11A1* [c.1647+61GT(10_13)] found no significant difference between the three cattle breeds included in this study. Our result differs from that of a previous study describing an increased number of genotypes for the MS1 microsatellite in *B. indicus* cattle breeds compared to *B. taurus* breeds (Hasenauer et al., 2013). Conflicting data also exists regarding the role this microsatellite plays in resistance to bTB. Indeed, whereas a study performed in African zebu cattle found that higher numbers of GT repeats in the MS1 microsatellite were associated with a reduction in traits indicative of bTB (Kadarmideen et al., 2011), a study using Argentine cattle found no association between different lengths of this microsatellite and response to the tuberculin skin test (Hasenauer et al., 2018). If the MS1 microsatellite in the 3′UTR of the bovine *SLC11A1* gene indeed plays a biological role in NRAMP1 expression, it is likely to be due to differences in post-transcriptional regulation of the mRNA, possibly by influencing mRNA stability, similarly as described for other molecules (Citores et al., 2004; Chen et al., 2007). Our attempt to investigate this using a luciferase reporter construct in an *in vitro* approach did not identify a significant difference in luciferase expression in either CHO or RAW264.7 cells, suggesting that different numbers of GT repeats in the *SLC11A1* MS1 microsatellite did not impact on NRAMP1 protein expression in this system. Whether the results would differ if an appropriate bovine macrophage cell line is employed remains to be seen.

## Conclusion

In conclusion, our study demonstrated that variations in the bovine *SLC11A1* gene are associated with a functional difference in the expression of NRAMP1 in bovine MDM. Since NRAMP1 is known to regulate the level of iron within phagosomes, this provides further evidence that *SLC11A1*/NRAMP1 might indeed influence the susceptibility to intracellular pathogens, and in particular *M. bovis.* However, bTB is a complex disease which is likely to be influenced by polymorphisms in multiple genes, particularly those related to the immune system and host-pathogen interactions.

## Data Availability Statement

All datasets generated for this study are included in the article/Supplementary Material.

## Ethics Statement

The use of animals for blood collection was approved by the Royal Veterinary College (RVC) Ethical Committee, which subsequently resulted in granting Home Office License Number PPL7009059.

## Author Contributions

AH, RG, CE, PM, CL, and M-CB performed the experiments. AH, RG, CE, and MS analyzed the data. TT, MZS, and TC provided the valuable reagents. AH wrote the manuscript. BV-R and DW designed the study and revised the manuscript. All the authors read and approved the final manuscript.

## Conflict of Interest

The authors declare that the research was conducted in the absence of any commercial or financial relationships that could be construed as a potential conflict of interest.

## Footnotes

1 https://www.ncbi.nlm.nih.gov/nuccore/NM_174652.2

2 https://www.ncbi.nlm.nih.gov/tools/primer-blast/

3 http://vassarstats.net/index.html

4 https://www.ncbi.nlm.nih.gov/nuccore/NM_174652.2

5 https://www.ensembl.org/Bos_taurus/Gene/Variation_Gene/Table?db=core;g=ENSBTAG00000015520;r=2:106392590-106403650
