# Supplementary material for: Analysis of Genetic Variation in the Bovine SLC11A1 Gene, Its Influence on the Expression of NRAMP1 and Potential Association With Resistance to Bovine Tuberculosis
Source: Front Microbiol. 2020 Jun 30;11:1420. doi: 10.3389/fmicb.2020.01420 (PMC7341946; doi:10.3389/fmicb.2020.01420)
Supplement: Supplementary file 1 [file Data_Sheet_1.docx]

**Supplementary Table 1. Primers for sequencing and cloning the bovine *SLC11A1* gene.**

| **Primer Name** | **Primer Sequence (5’-3’)** | **Annealing Temp. (°C)** | **Elongation Time (s)** |
| --- | --- | --- | --- |
| *SLC11A1* Coding 1 | FOR: TGCGGTCCTCATGTCAGGTG  REV: ATGTTGGCCTCTCGGATGTC | 65.5 | 30 |
| *SLC11A1* Coding 2 | FOR: GCATTCCTCCCTGGTCAAGTC  REV: TCATCCCGAGGTCCTCCCCTT | 65.5 | 30 |
| *SLC11A1* MS1 | FOR: AGGATCAGGAGAAGGGGAGGA  REV: CAGCTTCCAGAACTCCCTGT | 62.0 | 20 |
| *SLC11A1* MS2 | FOR: AAGGCAGCAAGACAGACAGG  REV: ATGGAACTCACGTTGGCTG | 62.0 | 20 |
| *SLC11A1* MS1 cloning | FOR: GCGTTTAAACTGGCCACGGGTGG  REV: CGTCTAGATGGCCAGCTTCCAGA | 62.0 | 30 |

**Supplementary Table 2. Polymorphisms identified in the coding region of the *SLC11A1* gene in Brown Swiss, Holstein-Friesian and Sahiwal cattle.**

| **Name** | **Exon** | **Position on chromosome 2** | **SNP ID** | **Alternative SNP ID** | **Major allele** | **Minor allele** | **SNP type** | **Amino acid change** |
| --- | --- | --- | --- | --- | --- | --- | --- | --- |
| SNP1 | 2 | 106393420 | c.87A>G | rs109614179 | A | G | Synonymous | n/a |
| SNP2 | 8 | 106397600 | c.650C>T | rs109915208 | C | T | Non-synonymous | Ala – Val |
| SNP3 | 10 | 106399875 | c.961G>A | rs109551090 | G | A | Non-synonymous | Asp - Asn |
| SNP4 | 11 | 106400121 | c.1066C>G | rs109453173 | C | G | Non-synonymous | Pro - Ala |
| SNP5 | 15 | 106403056 | c.1592G>C | rs110347562 | G | C | Non-synonymous | Arg - Pro |

**Supplementary Table 3. *SLC11A1* genotyping data for individual Brown Swiss, Holstein-Friesian and Sahiwal cattle.**

| **BREED** | **COW ID** | **POLYMORPHISMS** | | | | | |
| --- | --- | --- | --- | --- | --- | --- | --- |
|  |  | **87 A>G** | **650 C>T** | **961 G>A** | **1066 C>G** | **1592 G>C** | **MS1** |
| Brown Swiss | 116 | A/A | C/C | G/G | C/G | G/G | 11/12 |
| Brown Swiss | 119 | A/A | C/C | G/G | G/G | G/G | 11/12 |
| Brown Swiss | 1215 | A/A | C/C | G/G | C/G | G/G | 11/12 |
| Brown Swiss | 139 | A/A | C/C | G/G | C/G | G/G | 11/12 |
| Brown Swiss | 796 | A/A | C/C | G/G | C/C | G/G | 11/12 |
| Brown Swiss | Bloom | A/A | C/C | G/G | G/G | G/G | 10/10 |
| Brown Swiss | Dazzle | A/A | C/C | G/G | C/G | G/G | 11/12 |
| Brown Swiss | Janis | A/A | C/C | G/G | C/C | G/G | 11/12 |
| Brown Swiss | Samba | A/A | C/C | G/G | C/C | G/G | 12/12 |
| Brown Swiss | Stardust | A/A | C/C | G/G | C/C | G/G | 10/12 |
| Brown Swiss | Jo (809) | A/A | C/C | G/G | C/C | G/G | 11/12 |
| Brown Swiss | Linda | A/A | C/C | G/G | C/C | G/G | 11/12 |
| Brown Swiss | Letitia | A/A | C/C | G/G | C/C | G/G | 11/12 |
| Brown Swiss | Trixie | A/A | C/C | G/A | C/G | G/G | 11/12 |
| Brown Swiss | Joyce | A/A | C/C | G/G | C/C | G/G | 11/12 |
| Holstein Friesian | 1042 | A/A | C/C | G/G | C/C | G/G | 11/12 |
| Holstein Friesian | 1062 | A/A | C/C | G/G | C/C | G/G | 11/12 |
| Holstein Friesian | 1120 | A/A | C/T | G/G | C/G | G/G | 11/12 |
| Holstein Friesian | 1019 | A/A | C/C | G/G | C/C | G/G | 12/12 |
| Holstein Friesian | 742 | A/A | C/C | G/G | C/C | G/G | 11/12 |
| Holstein Friesian | 746 | A/A | C/C | G/G | C/C | G/G | 11/12 |
| Holstein Friesian | 883 | A/A | C/C | G/G | C/C | G/G | 12/12 |
| Holstein Friesian | 958 | A/A | C/C | G/G | C/C | G/G | 11/12 |
| Holstein Friesian | Beth | A/A | C/C | G/G | C/G | G/G | 11/12 |
| Holstein Friesian | Cherry | A/A | C/C | G/G | C/C | G/G | 11/12 |
| Holstein Friesian | Fable | A/A | T/T | G/G | G/G | G/G | 10/10 |
| Holstein Friesian | Montana | A/A | C/C | G/G | C/C | G/G | 11/12 |
| Holstein Friesian | 1127 | A/A | C/C | G/G | C/C | G/G | 11/12 |
| Holstein Friesian | 804 | A/A | C/C | G/G | C/C | G/G | 11/12 |
| Holstein Friesian | 931 | A/A | C/C | G/G | C/C | G/G | 11/12 |
| Sahiwal | PC001 | A/A | C/C | G/G | C/C | G/G | 11/12 |
| Sahiwal | PC002 | A/A | C/C | G/A | C/G | G/C | 11/12 |
| Sahiwal | PC003 | A/G | C/C | G/A | C/G | G/C | 11/12 |
| Sahiwal | PC004 | A/A | C/C | G/G | C/C | G/G | 11/12 |
| Sahiwal | PC005 | A/G | C/C | G/A | C/G | G/G | 10/11 |
| Sahiwal | PC006 | A/G | C/C | G/A | C/G | G/C | 11/12 |
| Sahiwal | PC007 | G/G | C/C | A/A | G/G | G/G | 11/12 |
| Sahiwal | PC008 | G/G | C/C | A/A | G/G | C/C | 11/12 |
| Sahiwal | PC017 | A/A | C/C | G/G | G/G | G/G | 11/12 |
| Sahiwal | PC018 | A/A | C/C | G/G | G/G | G/G | 11/12 |
| Sahiwal | PA001 | A/A | C/C | G/G | C/C | G/G | 11/12 |
| Sahiwal | PA008 | A/A | C/C | G/G | C/C | G/G | 11/12 |
| Sahiwal | PA006 | A/G | C/C | G/A | C/G | G/G | 10/12 |
| Sahiwal | PA007 | A/A | C/C | G/G | C/C | G/G | 11/12 |
| Sahiwal | PA002 | A/A | C/C | G/G | C/C | G/G | 11/12 |

**
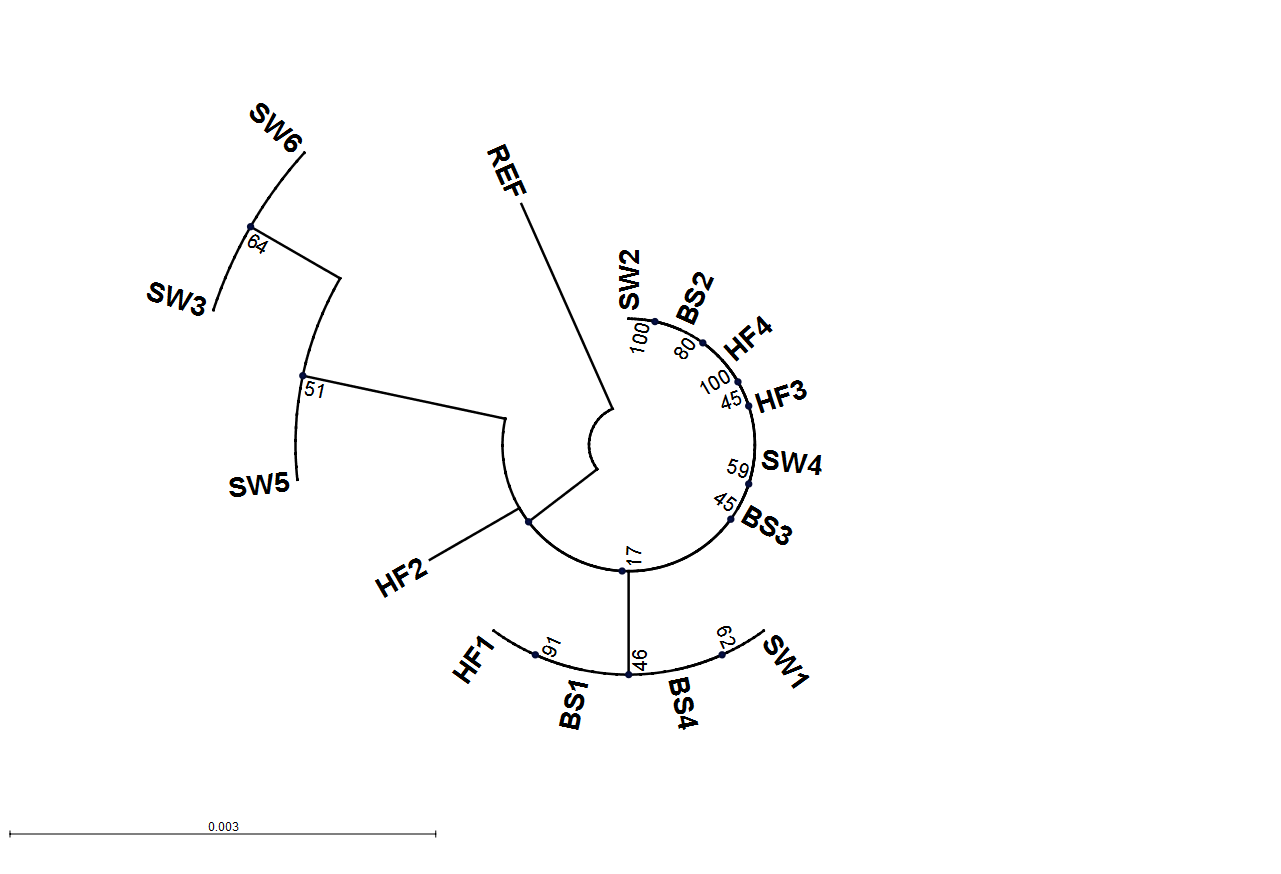
**

**Supplementary Figure 1. Phylogenetic tree of *SLC11A1* genotypes.** A maximum likelihood phylogenetic tree was constructed using the genotypes identified from sequencing the *SLC11A1* coding region in Brown Swiss, Holstein Friesian and Sahiwal cattle. The *B. taurus* *SLC11A1* reference sequence (<https://www.ncbi.nlm.nih.gov/nuccore/NM_174652.2>) was also included in the analysis. Bootstrap values (out of 100) are indicated at branch points. BS=Brown Swiss. HF=Holstein Friesian. SW=Sahiwal. REF=Reference.

| **CHO Cells** | **RAW 267.4 Cells** |
| --- | --- |
| 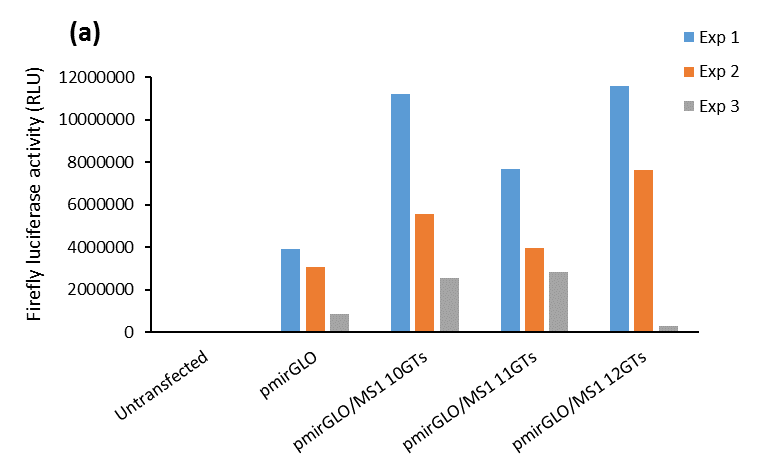 | 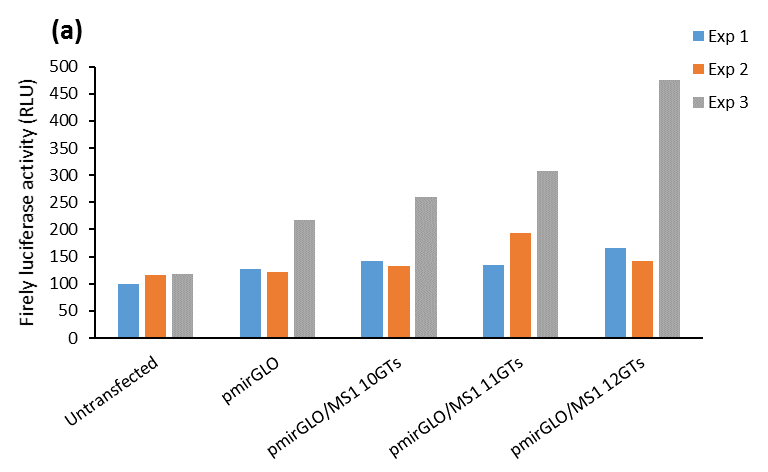 |
| 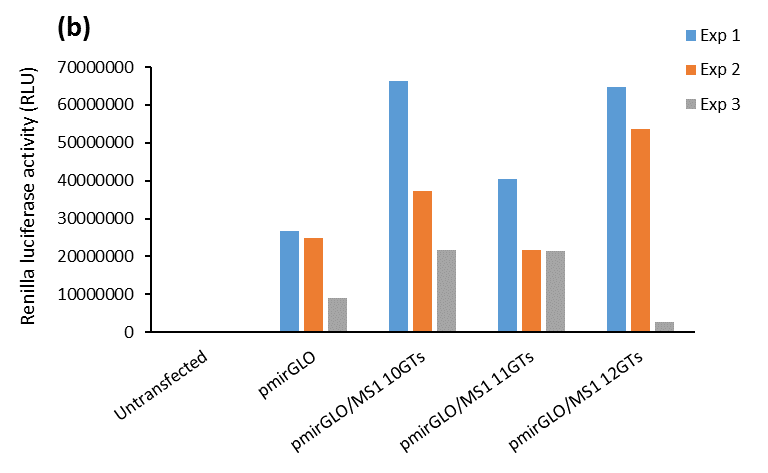 | 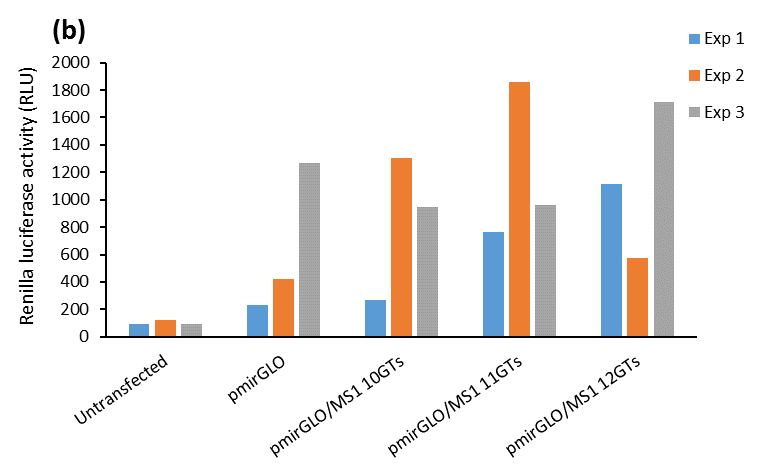 |
| 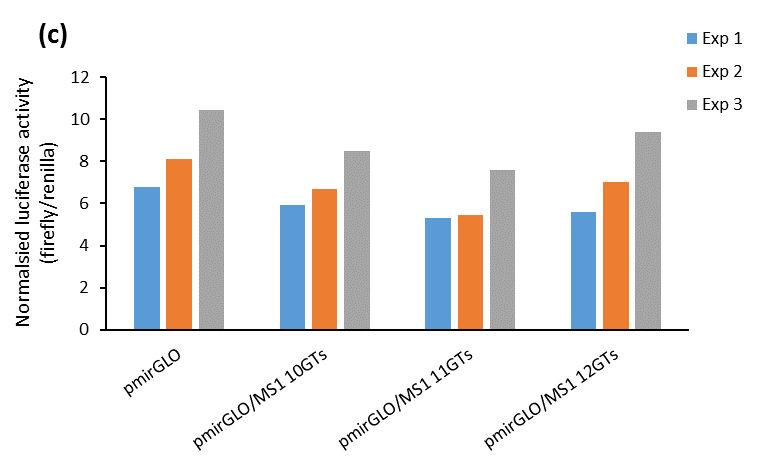 | 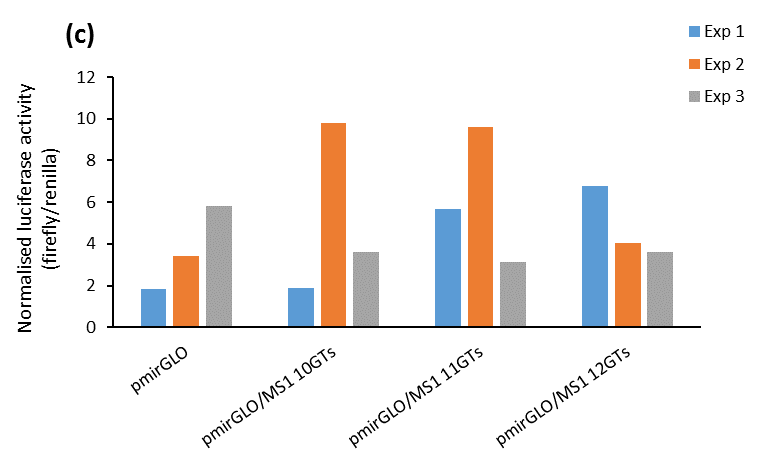 |

**Supplementary Figure 2. Assessment of (a) renilla, (b) firefly and (c) normalised luciferase activity in CHO and RAW 264.7 cells transfected with pmirGLO plasmids.** CHO and RAW 264.7 cells were transfected with pmirGLO constructs and after 24 hrs assayed for luciferase activity using the Dual-GLO luciferase assay system. The renilla luciferase activity was used to give a measure of transfection efficiency for the normalisation of gene expression, while the firefly luciferase activity allows the influence of the inserted sequence to be evaluated. Results of individual experiments are shown.
